# Pyoderma Gangrenosum–like Wounds in Leukocyte Adhesion Deficiency: Case Report and Review of Literature

**DOI:** 10.1097/GOX.0000000000001886

**Published:** 2018-08-08

**Authors:** Andrew M. Simpson, Karin Chen, John F. Bohnsack, Matthew N. Lamont, Faizi A. Siddiqi, Barbu Gociman

**Affiliations:** From the * Department of Surgery, Division of Plastic Surgery, University of Utah Health Sciences Center, Salt Lake City, Utah; † Division of Allergy and Immunology, Department of Pediatrics, University of Utah School of Medicine, Salt Lake City, Utah; ‡ Division of Allergy and Immunology, Division of Rheumatology, Department of Pediatrics, University of Utah School of Medicine, Salt Lake City, Utah.

## Abstract

Leukocyte adhesion deficiency (LAD) is a rare primary immunodeficiency characterized by impairment of leukocyte migration during an inflammatory response. LAD patients can experience recurrent neutrophilic wounds similar to pyoderma gangrenosum (PG), predominantly of the skin and mucosal surfaces. There have been only a few reports addressing the management of extensive, life-threatening wounds in LAD patients. We describe here both the systemic and local management employed to successfully treat a severe PG-like cutaneous lesion in the setting of LAD in a 9-year-old female. A comprehensive literature review was performed to identify previously reported similar cases. Under aggressive systemic and local management, the wound was stabilized and complete epithelialization was achieved in 8 months. Eight studies documenting 11 patients with LAD and PG-like lesions were identified in our review of the literature. The complexity of wounds associated with LAD requires an aggressive, multidisciplinary approach. Involvement of pediatrics, immunology, plastic surgery, infectious disease, and physical therapy is essential to obtaining a positive outcome. In the setting of LAD with PG-like lesions, the only viable option is allowing for closure by secondary epithelialization. This was achieved in our patient once the wound was stabilized with the systemic administration of infliximab and topical administration of tacrolimus.

## Abstract 2

Supplemental Digital Content is available in the text.

Leukocyte adhesion deficiency (LAD) is a rare primary immunodeficiency.^1^ It is characterized by impairment of the leukocyte migration from the blood stream into tissues that normally occurs in the setting of an inflammatory response.

LAD type I (LAD-1) is the most common subtype and is characterized by a deficiency in CD18 caused by mutations in integrin beta-2. It is further subclassified into mild/moderate and severe phenotypes, based on the expression level of CD18. The expression level is between 2–20% of normal in the mild/moderate phenotype, and less than 2% in the severe phenotype.^1^ Patients with the mild/moderate phenotype usually survive into adulthood. Typically, these patients experience recurrent bacterial infections, predominantly of the skin and mucosal surfaces. Minor injuries are associated with pronounced leukocytosis and cause wounds with markedly reduced healing capacity with possible progression to pyoderma gangrenosum (PG)–like lesions. PG-like wounds are similar clinically to classical PG, but lack the dermal neutrophilia visible on histology.^2^ Patients with the severe phenotype present with more frequent and severe wounds, often not surviving childhood.^3^

Although there is increasing interest in LAD, there is no consensus as to the efficacy of treatment modalities.^4,5^ We describe both the systemic and local management employed to successfully treat a severe cutaneous lesion in a child with LAD-1.

## CASE REPORT

**Fig. 1.**
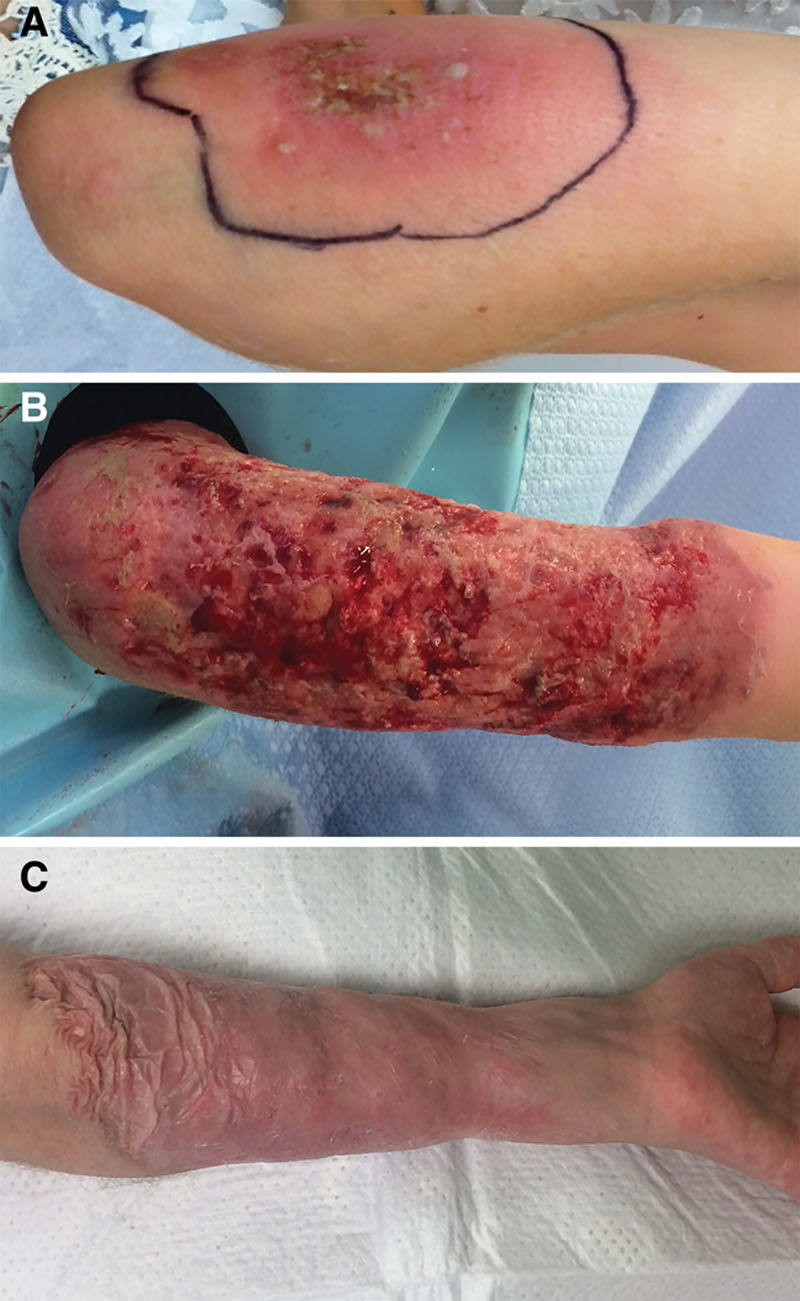
Wound appearance throughout the treatment process. A, Initial appearance of the wound on presentation, 1 week after minor abrasive trauma in a playground accident. B, Wound stabilization and healing with moderate hypergranulation tissue present in the wound bed, 3 months after initial injury. C, Healed wound with complete secondary epithelialization, 1 year after initial injury.

A 9-year-old female presented with progressive, PG-like lesions involving her left arm following a minor abrasive trauma in a playground accident (Fig. 1A). She had been previously diagnosed with LAD-1 at 18 months of age. Her CD18 expression level was 5–10%, or mild/moderate phenotype. She had never experienced life-threatening wounds. Her most recent clinical course involved 3.5 weeks of IV antibiotics and multiple wound debridement procedures at a peripheral site. Her lesions significantly expanded after debridement, consistent with PG-like wounds. She was then transferred to our tertiary center.

The patient was cared for by pediatrics (general medical care, care coordination), immunology (direction of immunosuppressive therapies) infectious disease (direction of empiric and culture-directed antibiotic and antifungal therapies) and plastic surgery (direction of wound care, debridement, and acquisition of biopsies). A punch biopsy was obtained from the central wound, histology demonstrated necrosis, focal superficial ulceration of the epidermis with mixed inflammatory infiltrate including histioctyes, multinucleated giant cells, lymphocytes, and focal neutrophils. There was a lack of dermal neutrophilia, consistent with PG-like disease. Systemic therapy consisting of prednisone (1 mg/kg/d), cyclosporine (goal trough level 100–200 ng/mL) and intravenous immunoglobulin (1 g/kg every 2 weeks) was initiated. Initially the wounds were treated empirically with broad spectrum antibiotics involving meropenem (500 mg IV q8h) and vancomycin (500 mg IV q12h). On day 104, a tissue culture obtained during debridement demonstrated deep infection with *Fusarium*, an invasive fungal infection typically occurring in immunocompromised patients. This corresponded to clinical worsening and elevation in C-reactive protein. She was treated initially with dual liposomal amphotericin (10 mg/kg/d IV) and voriconazole (9 mg/kg/dose IV q12), which was then stepped down to liposomal amphotericin alone (**see table, Supplemental Digital Content 1**, which displays Laboratory findings and medications during treatment course, http://links.lww.com/PRSGO/A819). This treatment continued until the wounds were closed. The wounds were initially dressed with SilvaSorb Site Dressing (Medline Industries Inc., Salt Lake City, Utah.) and changed every 3 days.

Despite this management, her wounds continued to worsen. Methylprednisolone (30 mg/kg IV weekly) was added to the treatment regimen after 3 weeks. During dressing changes, the wounds were meticulously handled and only frankly necrotic or loose hypergranulation tissue was debrided to mitigate the risk of secondary infection. Sargramostim (Sanofi US, Bridgewater, N.J.), a recombinant granulocyte-macrophage colony-stimulating factor, was applied topically at each dressing change.^4^

The dressing was substituted with Aquacel Ag (ConvaTec, Bridgewater, N.J.), an antimicrobial hydrofiber dressing, and this dressing was changed twice per week to limit the amount of wound manipulation. Shortly thereafter, IV infliximab infusions (10 mg/kg every 2 weeks), a tumor necrosis factor blocker and topical tacrolimus, a macrolide calcineurin inhibitor, were added to the regimen. Within days, the wounds had stabilized, and granulation tissue became apparent. Epithelialization was observed at all edges of the wound bed. As wound healing progressed, hypergranulation tissue was noted (Fig. 1B). Cautious debridement of the hypergranulation tissue with removal of necrotic tissue along with frequent infection monitoring was required to allow for complete epithelialization of the wound.

The dressing change frequency was changed from twice weekly to weekly, as healing progressed. Complete epithelialization of the wound was accomplished in 8 months (Fig. 1C). Aggressive physiotherapy was employed throughout the healing process to avoid the development of contractures. Full range of motion was maintained. The wound was observed and stable for 2 years postoperatively.

## REVIEW OF LITERATURE

A literature search was performed for the period 1985–2018 using the PubMed database National Center for Biotechnology Information, National Library of Medicine (Bethesda, Md.). Keywords used were: “leukocyte adhesion deficiency,” “pyoderma gangrenosum,” “infection,” and “surgery.”

## DISCUSSION

Multiple immunosuppressive modalities have been employed in the setting of LAD. In the management of this patient, wound healing was only seen after the systemic administration of infliximab and topical administration of tacrolimus. Due to the concomitant administration of these 2 medications, we cannot discern which one was responsible for the improvement seen. It is unclear why these therapies are beneficial in patients with LAD; as targeted biologic medications become better understood, it is hoped that mechanisms of action and best practice guidelines for these modalities can be developed.^5^ It is possible that topical administration of tacrolimus in our situation increased the local concentration of drug at the site, bolstering its effect. Due to a lack of best practice guidelines, the treatment decisions taken in this case were based on literature reviews, expert opinion, and a graduated level of potency in terms of immunosuppressive medication as milder therapies failed.

Our literature review demonstrated 11 patients in 8 studies. All patients were managed with combination local and systemic therapy. One patient died due to systemic fungal infection (Table 1).^2^ The variety of treatment modalities and medications used highlights the complexities and lack of evidence-based guidelines in this difficult patient population. These studies were used to assist in the guidance of clinical decision making in the present case.

**Table 1.**
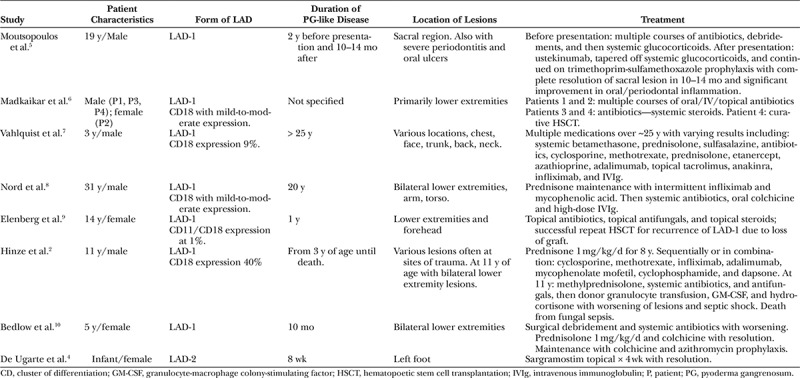
Literature Review of Patients with LAD Presenting with Pyoderma Gangrenosum–like Lesions and Their Subsequent Treatment and Outcome

Extensive, full-thickness cutaneous wounds are traditionally managed with surgical reconstruction. These modalities include skin grafts, local, regional, or free flaps. In the setting of PG-like disease, none of these options can be applied, as any disruption of tissue can have devastating consequences due to pathergy. It is critical to have a frank discussion with these patients and their families about the importance of patience and diligent wound care. To allow for secondary epithelialization of such large wounds, meticulous local wound care and aggressive physical therapy are essential. Only very cautious, conservative debridement of frankly necrotic or loose hypergranulation tissue should be performed with an aim of managing (in this case, *Fusarium* infection) or decreasing the risk for secondary infection. Our wound dressing recommendation based on this experience is an antimicrobial hydrofiber. This dressing provides local antimicrobial therapy and appropriate moisture control, allowing for epithelialization while remaining in place undisturbed between dressing changes.

Successful treatment of PG-like wounds in the setting of LAD requires a multidisciplinary effort from immunology, infectious disease, pediatrics, physiotherapy, and plastic surgery. Early recognition of the disease may prevent paradoxical worsening following attempts at wound debridement. The differential diagnosis of PG-like wounds include infection, vasculitis, malignancy, and inflammatory conditions such as hidradenitis. Diagnosis is made on the basis of history, clinical features, and tissue biopsy consistent with PG but lacking dermal neutrophilia. It is our hope that with continued reporting of management strategies in this patient population, clinical practice guidelines may be developed.

## CONCLUSIONS

Severe cutaneous lesions in LAD patients can be successfully managed with specific systemic immunosuppression and personalized local wound care. Future prospective studies examining targeted immunosuppressive regimens could lead to best practice guidelines. Plastic surgeons should be aware of these difficult wounds in this patient population and should avoid interventions that may lead to worsening.

## ACKNOWLEDGMENTS

This article is exempt from institutional review board designation by nature of being a case report. This work complies with the Declaration of Helsinki ethics standard.

## Supplementary Material
