# Supplementary material for: Pyoderma Gangrenosum–like Wounds in Leukocyte Adhesion Deficiency: Case Report and Review of Literature
Source: Plast Reconstr Surg Glob Open. 2018 Aug 8;6(8):e1886. doi: 10.1097/GOX.0000000000001886 (PMC6143322; doi:10.1097/GOX.0000000000001886)
Supplement: Supplementary file 1 [file gox-6-e1886-s001.pdf]

Supplementary Table 1: Laboratory findings and medications during treatment course, day 0 refers to the day the patient presented to the peripheral hospital site, 3.5 weeks prior to presentation to tertiary care.

**Hospitalization day** (normal lab values)	**Day 0**	**Day 44**	**Day 62**	**Day 93**	**Day 104**	**Day 109**	**Day 111**	**Day 279** Wounds Healed	**Day 358**
Absolute neutrophil count (4500-13500 cells/μL)	24000	47400	15500	63900	43600	36400	ND	36500	22400
C-reactive protein (<0.8mg/dL)	15.9	16.7	1.3	27	>32.0	12.6	1.7	7.7	1.4
IL-6 (<5pg/mL)	ND	50	ND	800	19	ND	ND	ND	ND
TNF-α (<5pg/mL)	ND	<5	ND	<5	<5	ND	ND	ND	ND
Soluble IL-2 receptor (<1033 pg/ml)	ND	454	ND	427	1028	ND	ND	ND	ND
**Infections**	-	-	-	*Rhinovirus* URTI	*Fusarium* Cellulitis	-	-	-	-
**Antimicrobial medications**									
Meropenem (500 mg IV q8h)	+	+	+	+	-	-	-	-	-
Vancomycin (500 mg IV q12h)	+	+	+	+	-	-	-	-	-
Liposomal amphotericin (10 mg/kg/day IV)	-	-	-	-	+	+	+	+	-
Voriconazole (9 mg/kg/dose IV q12)	-	-	-	-	+	+	-	-	-
**Immunomodulatory medications**									
Prednisone (1mg/kg/day)	-	taper	taper	-	-	-	-	-	-
Cyclosporine (goal trough level 100-200 ng/mL)	-	+	+	+	+	+	+	-	-
Methylprednisolone (30mg/kg IV weekly)	-	+	+	+	+	+	+	-	-
High-dose IGIV (1g/kg every two weeks)	-	+	+	+	+	+	+	-	-
Infliximab (10 mg/kg every 2 weeks)	-	-	+	+	+	+	+	-	-
**Topical medications**									
GM CSF	-	+	+	+	+	+	+	-	-
Clobetasol	-	+	-	-	-	-	-	-	-
Tacrolimus	-	-	+	+	+	+	+	-	-

IGIV = immune globulin intravenous; ND = not done; URTI = upper respiratory tract infection; IL = interleukin; TNF = tumor necrosis factor; GM CSF = granulocyte-macrophage colony-stimulating factor
